# METTL16 inhibits papillary thyroid cancer tumorigenicity through m^6^A/YTHDC2/SCD1-regulated lipid metabolism

**DOI:** 10.1007/s00018-024-05146-x

**Published:** 2024-02-09

**Authors:** Qiang Li, Yaju Wang, Xiangshu Meng, Wenjing Wang, Feifan Duan, Shuya Chen, Yukun Zhang, Zhiyong Sheng, Yu Gao, Lei Zhou

**Affiliations:** 1 Department of Cell Biology, School of Life Science, Bengbu Medical College, Bengbu, Anhui China; 2 Anhui Province Key Laboratory of Translational Cancer Research, Bengbu Medical College, Bengbu, Anhui China; 3 Department of Biotechnology, School of Life Science, Bengbu Medical College, Bengbu, Anhui China; 4 Bengbu Medical College Key Laboratory of Cancer Research and Clinical Laboratory Diagnosis, Bengbu Medical College, Bengbu, 233030 China; 5 Guangxi Academy of Medical Sciences, the People’s Hospital of Guangxi Zhuang Autonomous Region, Nanning, 530021 China

**Keywords:** METTL16, PTC, Lipid metabolism, RNA m^6^A methylation, Targeted therapy

## Abstract

Papillary thyroid carcinoma (PTC) stands as the leading cancer type among endocrine malignancies, and there exists a strong correlation between thyroid cancer and obesity. However, the clinical significance and molecular mechanism of lipid metabolism in the development of PTC remain unclear. In this study, it was demonstrated that the downregulation of METTL16 enhanced lipid metabolism and promoted the malignant progression of PTC. METTL16 was expressed at lower levels in PTC tissues because of DNMT1-mediated hypermethylation of its promoter. Loss- and gain-of-function studies clarified the effects of METTL16 on PTC progression. METTL16 overexpression increased the abundance of m^6^A in SCD1 cells, increasing RNA decay via the m^6^A reader YTHDC2. The SCD1 inhibitor A939572 inhibited growth and slowed down lipid metabolism in PTC cells. These results confirm the crucial role of METTL16 in restraining PTC progression through SCD1-activated lipid metabolism in cooperation with YTHDC2. This suggests that the combination of METTL16 and anti-SCD1 blockade might constitute an effective therapy for PTC.

**Supplementary Information:**

The online version contains supplementary material available at 10.1007/s00018-024-05146-x.

## Introduction

The incidence rate of thyroid cancer worldwide has increased, as thyroid cancer has become the most prevalent endocrine tumor [1]. Papillary thyroid carcinoma (PTC) is the most common subtype of thyroid cancer [2]. Despite the favorable prognosis for the majority of patients, some PTC patients display locoregional recurrence and aggressive clinicopathological characteristics, such as compression of adjacent organs, invasion of the thyroid capsule, and dissemination to nearby lymph nodes [3]. Additionally, the management of central neck nodes in PTC patients remains a contentious issue in PTC treatment, as lymph node metastasis is prevalent and can impact survival and recurrence rates positively [4]. Consequently, there is an urgent need for the identification of novel molecular markers for PTC diagnosis, assessment of metastasis, and targeted therapy.

In eukaryotic cells, RNA is always accompanied by N6-methyladenosine (m^6^A) methylation [5]. The m^6^A modification is introduced into mRNA by “writers” (METTL3, METTL14, and WTASP) [6] and eliminated by “erasers” (FTO and ALKBH5) [7]. The reader proteins (YTHDF1-3, YTHDC1-2, IGF2BP1-3, and EIF) identify the m^6^A modification [8] and attach to the m^6^A motif to directly or indirectly govern RNA functions, including stability, nuclear processing, primary microRNA processing, and RNA‒protein interactions [9]. Dysfunction of m^6^A modulators could influence multiple cellular processes, including tumorigenesis [10]. METTL16, a newly discovered m^6^A methyltransferase, is also associated with various malignant characteristics in different cancers [11] and exhibits reduced expression levels in PTC tissues [12]. Nonetheless, the possible function of METTL16 as a writer of m^6^A RNA in PTC remains unclear.

Cancer cells may reprogram their energy metabolism to support the initiation and progression of malignant tumors [13]. Elevated free fatty acid (FFA) synthesis is one of the key features of metabolic reprogramming in many cancer types, as cancer cells grow widely and demand great and long-term supplementation of FFAs for energy production, protein modification and membrane biosynthesis [14]. Abnormal lipid metabolism is one of the contributors to obesity, and a prior investigation demonstrated a positive correlation between obesity and the occurrence of thyroid cancer [15]. In addition, emerging evidence suggests that many RNA m^6^A regulators, such as METTL3, METTL14 and FTO, are involved in lipid metabolism in cancers [16]. Accordingly, elucidating the relationship between METTL16 and lipid metabolism in thyroid cancer will aid in understanding the pathogenesis of PTC and lead to novel treatment strategies for this disease.

Here, this research investigated the biological role of METTL16/YTHDC2/SCD1 in regulating lipid metabolism in PTC progression and proposed that METTL16 and its downstream regulatory molecules could be new PTC diagnostic markers and therapeutic targets for PTC.

## Materials and methods

### Patient samples

The neoplastic tissues and adjacent peritumoral tissues were collected from 58 PTC patients at the Department of Thyroid and Breast Surgery, The First Affiliated Hospital of Bengbu Medical College (Bengbu, China). Permission for this study was granted by the Review Board of Bengbu Medical College (Reference no. LKPZ074[2021]).

### Immunohistochemistry (IHC)

Tissue microarray (TMA) and immunohistochemistry (IHC) were conducted by Shanghai Outdo Biotech. (Shanghai, China). Briefly, paraffin-embedded tissues were blocked with reagents that could block peroxidase activity (anhydrous methanol: H_2_O_2_: ddH2O = 3.2: 1: 0.8). Then, antibodies against METTL16 (1:400; ab252420; Abcam) or SCD1 (1:400; ab236868; Abcam) were added and incubated at 4 °C overnight. The slices were rinsed and cultured with HRP-conjugated antibodies (Agilent DAKO) on day two, followed by diaminobenzidine and hematoxylin staining at room temperature.

### Bioinformatics analysis

The gene expression and clinical information of PTC patients were obtained from The Cancer Genome Atlas (TCGA) database (https://cancergenome.nih.gov/) and cBioPortal (https://www.cbioportal.org/). DNA methylation status was assessed using MethPrimer (http://www.urogene.org/methprimer/). Predictions for RNA methylation were made using SRAMP (http://www.cuilab.cn/sramp).

### Cell culture

The BCPAP, TPC1, and K1 cells were procured from BNCC Company (Beijing, China). Authentication through DNA fingerprinting was also conducted. All cells were cultured in a CO_2_ incubator (37 °C, 5% CO_2,_ and 95% humidity) supplemented with DMEM (10% FBS, penicillin‒streptomycin antibiotics).

### RNA isolation, quantification and mRNA stability assays

An RNA extraction kit (R0017, Beyotime) was used for RNA isolation. Approximately 2 µg of RNA was reverse-transcribed to cDNA (D7170, Biyotime). The RNA concentrations were quantified by an Applied Biosystems 7500 instrument with RealStar Green Fast Mixture (A301, GenStar). β-Actin was used as the endogenous control. The primers used can be found in Supplementary Table 1. To assess mRNA stability, the transcriptional inhibitor actinomycin D (MCE, HY-17,559) was employed at a concentration of 5 µg/mL. After incubation with PTC cells for 0, 180, or 360 min, RNA was isolated and subjected to qRT-PCR for RNA stability detection. The loading control was HPRT1 because there were no m^6^A modifications in its mRNA.

### RNA m^6^A quantification and m^6^A dot blot assay

For RNA m^6^A measurements, 200 ng of RNA was bound to a m^6^A antibody and incubated for 1 h. Following three washes, m^6^A levels were measured at an absorbance of 450 nm using the EpiQuik m^6^A Kit (P-9005, Epiquik) and calculated based on the standard curve. For the m^6^A dot blot assay, 100 ng of mRNA was applied to an N^+^ membrane (GE Healthcare). After UV crosslinking (120 mJ/cm^2^ at 254 nm), blocking, and incubation with 1:1000 diluted m^6^A antibody (202,003, SYSY) and IgG-HRP (Beyotime, China), the membranes were incubated with a BeyoECL Plus Kit (Beyotime, China) for exposure and then imaged using a ChemiDoc XRS system (Bio-Rad). The loading control was methylene blue (MB) staining.

### Plasmid construction, transfection and promoter activity assays

To construct plasmids, the CDS of METTL16 wild type (GeneID: NM_024086.4), the CDS of DNMT1 (Gene ID: NM_001130823.3), and the METTL16 mutant (containing catalytically inactive mutants, PP185/186AA, which impair METTL16’s enzymatic activity) were synthesized by Sangon Biotech (Shanghai, China) and integrated into pcDNA 3.1 (+). The siRNA employed was designed and produced by RiboBio (Guangzhou, China). The Lipo8000™ transfection reagent (Beyotime, China) was used to facilitate transfection.

To construct lentiviral vectors, the coding sequences of METTL16-WT or METTL16-Mut were generated and inserted into the pHBLVCMVIE-IRES-Puro vector (HANBIO Biotechnology, Shanghai). The interference fragments were generated and inserted into the pHBLV-U6-puro vector (HANBIO Biotechnology, Shanghai). The 293T cells were used for virus packaging, and the stably transfected BCPAP and K1 cells were selected with puromycin (0.6 µg/mL).

For promoter activity assays. There were three predicted CpG islands in the promoter region of METTL16: the first (1313–1473 bp), the second (1513–1630 bp) and the third (1813–1942 bp). The full promoter regions (2000 nucleotides) of METTL16 (wild type, WT) and of the three other mutants (Mut1 with the third CpG island deleted, Mut2 with the third and second CpG island deleted, and Mut3 with the all of the three CpG islands deleted) were synthesized and subcloned and inserted into the PGL 3.0 basic vector by Sangon Biotech. The vectors were co-transfected with pcDNA 3.1 (+) empty vector or the DNMT1 overexpression vector into BCPAP or K1 cells with Lipo8000 Transfection Reagent (Beyotime). After 48 h of transfection, luciferase activity was determined using a luciferase reporter kit (FR201, Transgene). Firefly luciferase was used as an internal control.

### DNA methylation analysis

Before the transcription start site (2000 nucleotides), the potential CpG islands were predicted by MethPrimer. The data showed that the CpG island was located between − 1312 and − 1472 bp and between − 1512 and − 1629 bp upstream of the transcription start site. An EpiTect Fast DNA Bisulfite Kit (59,826, QIAGEN) was used for the bisulfite sequencing PCR (BSP) assay. Briefly, 100 ng of DNA was added to bisulfite reaction mixture, which was subsequently mixed thoroughly. Then, the bisulfite DNA was subjected to PCR in a thermal cycler (95 °C, 5 min; 60 °C, 10 min; 95 °C, 5 min; 60 °C, 10 min) and purified for subsequent BSP sequencing.

For the MSP (methylation-specific PCR) assay, genomic DNA from K1 and BCPAP cells transfected with the control or DNMT1 overexpression vector was obtained with a TIANamp Genomic DNA Kit (DP304, Tiangen). The Bisul Modification Kit (P-1026, Epiquik) was subsequently used for DNA bisulfite conversion. Subsequently, the converted DNA was subjected to methylation-specific PCR (MSP) using the MS-qPCR Fast Kit (P-1028, Epiquik). The primers utilized are listed in Supplementary Table 1. All the experimental procedures mentioned above were carried out in accordance with the manufacturer’s instructions.

### Chromatin immunoprecipitation (ChIP) assay

A ChIP Assay Kit (P2078, Beyotime) was used for this experiment. Briefly, formaldehyde (1%) was used for cell crosslinking. The cell lysate was sonicated to yield DNA fragments ranging from 200 to 1000 bp in size while placed in an ice-water mixture. A 10% input was retained for analysis, and the rest of the sample underwent immunoprecipitation using either an anti-DNMT1 antibody (2 µg, ab79822, Abcam) or an anti-5-mC antibody (2 µg, ab10805, Abcam). The negative control utilized IgG. After DNA purification with phenol chloroform, the samples were subjected to qPCR for subsequent calculations.

### RNA immunoprecipitation assay

K1 or BCPAP cells stably overexpressing METTL16 or with METTL16 knocked down and their corresponding control cells were crosslinked by UV (260 nm, 130 mJ/cm^2^) and harvested in cold PBS. RNA immunoprecipitation (RIP) was performed using a Magna RIP Kit (17–700, Millipore). Briefly, harvested cells were lysed (10% for input) and incubated with an anti-YTHDC2 antibody (1:1000; ab220160; Abcam) overnight at 4 °C. After washing, the immunoprecipitated complex was digested with proteinase K. RNA was extracted, detected via qRT‒PCR and normalized to the input. For m^6^A RNA binding experiments, total RNA from K1 cells stably overexpressing METTL16 or with METTL16 knocked down was treated with deoxyribonuclease I (Solarbio, China). The RNAs were sonicated and precipitated with Protein G Magnetic Beads (S1430S, NEB) bound to a m^6^A antibody (202,003, SYSY). After proteinase K (10 µg/mL) enzymolysis, RNAs were isolated for qRT‒PCR analysis (the input served as a control). For m^6^A sequencing, m^6^A-RNAs were acquired from the aforementioned m^6^A-RIP assay. The RNA libraries were created via quality inspection and subsequently subjected to analysis on an Illumina HiSeq instrument. The peaks were visualized with IGV software.

### Western blot analysis

Tissue or cell protein was obtained using RIPA lysis buffer (1% PMSF). Total protein (30 µg) was separated via SDS‒PAGE and subsequently transferred to a PVDF membrane. Skim milk (5% in TBST) was used for blocking. After three times of rinse, the membrane was washed and incubated overnight at 4 °C with antibodies (1:1000) against METTL16 (ab252420, Abcam), DNMT1 (ab188453, Abcam), SCD1 (ab236868, Abcam), and β-actin (66,009, Protein Tech, China), which were used as the normalization controls. The next day, the membranes were incubated with HRP-conjugated secondary antibodies (Beyotime, China). The membranes were subsequently exposed with a BeyoECL kit (Beyotime, China), after which images were acquired with a ChemiDoc XRS (Bio-Rad).

### Proliferation assay

The cells were seeded at a density of 1000 cells per well, and their viability was assessed every 24 h by measuring absorbance at 450 nm using a CCK-8 kit (Beyotime, China). For colony formation, 1000 transfected cells were reseeded into plates and cultured for 2 weeks. After fixation with 4% paraformaldehyde, the cells were stained with 0.2% crystal violet. For the EdU incorporation assay, 20,000 cells were seeded for transfection. EdU signals were detected with an EdU kit (C0081, Beyotime). Briefly, cells were cultured with EdU (10 µM) for 2 h. Then, the cells were rinsed and permeabilized with Triton X-100 (0.3%) for further reaction with click buffer (with the kit provided). Images were obtained, and the intensities were calculated with ImageJ software.

### Flow cytometry analysis

The percentage of apoptotic PTC cells was analyzed through flow cytometry. PTC cells were collected in binding buffer (200 µL) and dyed with propidium iodide (PI, 10 µL) and Annexin V-FITC (10 µL). After incubation, the cells were analyzed using a Beckman Coulter CytoFLEX flow cytometer.

### Cell mobility assay

For wound healing assays, transfected cells were reseeded and allowed to grow to confluence. A scratch was made in the middle of each well, and images were captured at 0 and 48 h. The migration distance was calculated using the formula: ((gap area [0 h] - [48 h])/[0 h]). In the Transwell assays, membranes were either coated with or without Matrigel. Subsequently, 30,000 transfected cells were loaded into the upper chambers, and the cells on the underside were counted after staining with crystal violet 48 h later.

### Fatty acid determination and TG content assay

The thawed samples were combined with tubes preloaded with a mixture of water, phosphoric acid (15%), and 4-methyl valeric acid (5 µg/mL). After homogenization and subsequent centrifugation, the supernatants were analyzed using GC‒MS (Agilent Model 7890 A/5975 C), and the concentration was determined based on the calibration curve.

### TG content assay and oil red O staining

Transfected PTC cells were cultivated in a medium containing 100 µM oleic acid (OA) for 48 h. The cell lysates were incubated with reagents (37 °C for 30 min) from a TG Assay Kit (E1003, Applygen, China) before TG analysis. For Oil Red O staining, after incubation with oleic acid, the cells were fixed (4% paraformaldehyde), stained with Oil Red O (Solarbio, China) and counterstained with DAPI. Images were captured for subsequent analysis.

### Luciferase activity assays

The wild-type (WT) or mutant (Mut) sequences of the SCD1 3’ UTR were subcloned and inserted into the pmirGlo dual luciferase reporter from Sangon Biotech (Shanghai, China). These two vectors were cotransfected with METTL16 overexpression vectors or negative controls into cells. After 48 h, a reporter assay kit (FR201, Transgene, China) was used to measure the relative luciferase activities, which were normalized to the firefly luciferase activity.

### Fatty acid oxidation assay

The rate of fatty acid oxidation was assessed by quantifying the levels of ^14^C-labeled acid-soluble metabolites (ASM, indicative of incomplete oxidation) and the release of ^14^CO_2_ (indicative of complete oxidation), as described previously [17]. In brief, 0.5 µCi/mL of (1–14 C)-palmitate (NEC534050UC, Perkin Elmer, Inc., USA), 0.15 mM palmitate, and 80 µM fatty acid-free BSA were introduced into the culture medium. After K1 cells were incubated with the METTL16 overexpression vector or METTL16 siRNA for 5 h, the medium was collected into a tube. Then, 500 µL of 3 M perchloric acid was added to facilitate CO_2_ release. The released CO_2_ was captured by covering the top of the tube with Whatman filter paper soaked with 0.1 M NaOH. The excess medium was centrifuged at 14,000 rpm for 15 min to eliminate particulate matter. The radioactivity in ASM (the culture media supernatants) and the CO_2_ concentration captured by the filter papers were calculated by a scintillation counter and normalized to the protein concentration.

### Animal experiment

Male nude BALB/C mice, aged 5 weeks, were acquired from Chang Zhou Cavens Laboratory Animal Ltd. These mice were provided with unrestricted access to both water and food, following a 12-h light and 12-h dark cycle. Subcutaneous injections of stably transfected K1 cells (2 × 10^6^ cells per mouse) were administered in the armpits of the mice. Once the tumors became palpable, their volume was measured every three days and calculated using the formula: length × width^2^ × 0.5. The tumor weight was detected after the mice were sacrificed.

For drug treatment, K1 cells with or without stable METTL16 knockdown were subcutaneously inoculated. When the tumors became palpable, the mice were subjected to treatment with either a vehicle alone or A939572 (100 mg/kg) every two days for a duration of 14 days, after which the treatment was discontinued. The remaining procedures were the same as those described previously.

For metastasis, 100 µL of cell suspension (2 × 10^6^ cells) was injected via the tail vein. The metastatic lymph nodes were enumerated and subjected to H&E staining. The Institutional Animal Care and Use Committee of Bengbu Medical College (Reference no. 2021LDK141) approved the animal experiments, and all procedures adhered to institutional guidelines.

### Statistical analysis

The data are expressed as the mean ± SEM. For two-group comparisons, Student’s t test was used, and for comparisons of multiple groups, one-way ANOVA with the Newman‒Keuls multiple comparison test was utilized. All the experiments were replicated at least three times. *P* < 0.05 indicated significance, and the asterisks were denoted as follows: *P < 0.05, **P < 0.01, and ***P < 0.001.

## Results

### METTL16 downregulation indicates poor prognosis in PTC patients

**Fig. 1:**
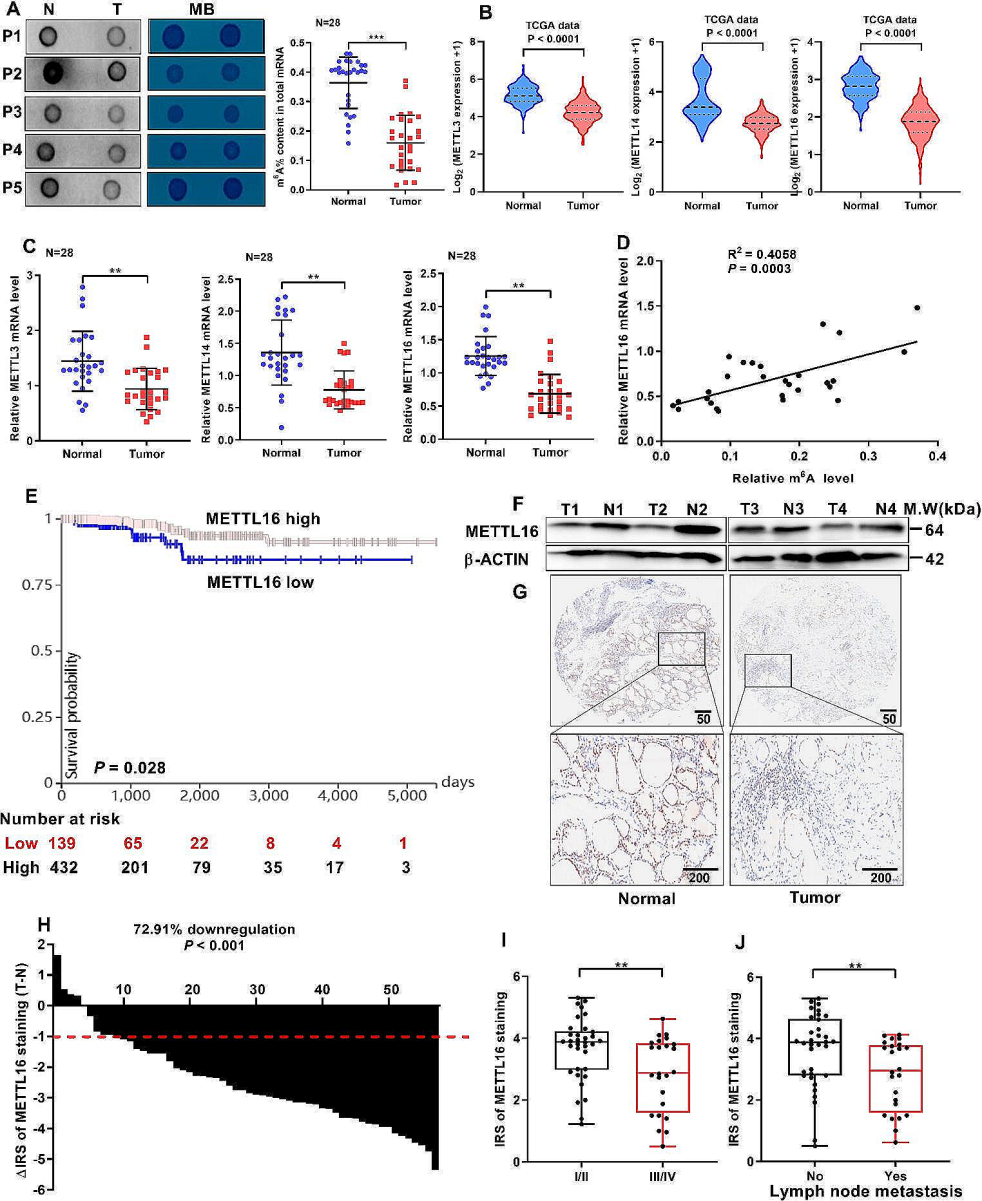
METTL16 downregulation indicates poor prognosis in PTC patients. (**A**) The m^6^A levels in tumor and paired tissues were measured via a m^6^A ELISA kit and a dot blot assay (*n* = 28). RNAs stained with methylene blue (MB) were used as loading controls (representative images in the right panel). (**B**) METTL14, METTL3, and METTL16 expression in normal thyroid tissues (*n* = 178) and PTC tissues (*n* = 493) in the TCGA cohort. (**C**) Levels of METTL3, METTL14, and METTL16 mRNA in paired PTC tissues (*n* = 28). (**D**) Relationships between m^6^A and METTL16 mRNA levels in PTC tissues (*n* = 28). (**E**) Kaplan‒Meier survival curves of patients in the TCGA cohort were analyzed using the online bioinformatics tool UCSC Xena (https://xena.ucsc.edu/). (**F**) METTL16 protein levels in paired PTC tissues were measured via western blotting. (**G**) IHC staining of METTL16 in paired tissues. Scale bars = 50 or 200 μm. (**H**) The immunoreactivity score (IRS) was calculated (△IRS = IRST - IRSN); an IRS value < − 1 indicated that METTL16 mRNA was downregulated in tumors (*n* = 58). (**I**) The expression pattern of METTL16 in stage I/II (*n* = 34) and stage III/IV PTC tissues (*n* = 24). (**J**) METTL16 expression in nonmetastatic (*n* = 34) and lymph node metastatic PTC tissues (*n* = 24). The data are presented as the means ± SEMs and were analyzed using Student’s t test or one-way ANOVA. *p < 0.05, **p < 0.01, ***p < 0.001

To determine the role of m^6^A levels in papillary thyroid carcinoma (PTC), the m^6^A RNA levels in 28 PTC tissues and paired adjacent tissues were systematically analyzed. The data showed that m^6^A levels were decreased in PTC tissues (Fig. 1A). These results were further verified using a m^6^A ELISA (Fig. 1A). Next, the expression patterns of regulators involved in m^6^A modification were analyzed. Analysis of the TCGA database showed that in addition to METTL3 and METTL14, the expression of the novel “m^6^A writer” METTL16 was also decreased in tumor tissue (Fig. 1B). In addition, METTL16 was positively correlated with METTL14 (Fig. S1A-C). The expression pattern was also similar to that observed via qRT‒PCR (Fig. 1C). Moreover, the METTL16 expression level was positively correlated with the m^6^A RNA level in the 28 PTC tissues. Hence, METTL16 was chosen for further study. In addition, PTC patients with low METTL16 expression had lower survival rates than did those with high METTL16 expression, as determined by the online tool Kaplan‒Meier plotter (Fig. 1E). Consistent with these findings, METTL16 protein levels were reduced in PTC tissues compared to normal tissues (Fig. 1F). The immunohistochemistry (IHC) staining results obtained from the tissue microarray also revealed a significant decrease in METTL16 expression in PTC tissues (Fig. 1G-H). Furthermore, the expression levels of METTL16 were lower in stage III/IV and metastatic tumors compared to stage I/II and nonmetastatic tumors (Fig. 1I-J). Taken together, these observations suggest that the downregulation of METTL16-mediated m^6^A modification may initiate PTC progression.

### DNMT1-mediated promoter hypomethylation restrains METTL16 expression

**Fig. 2:**
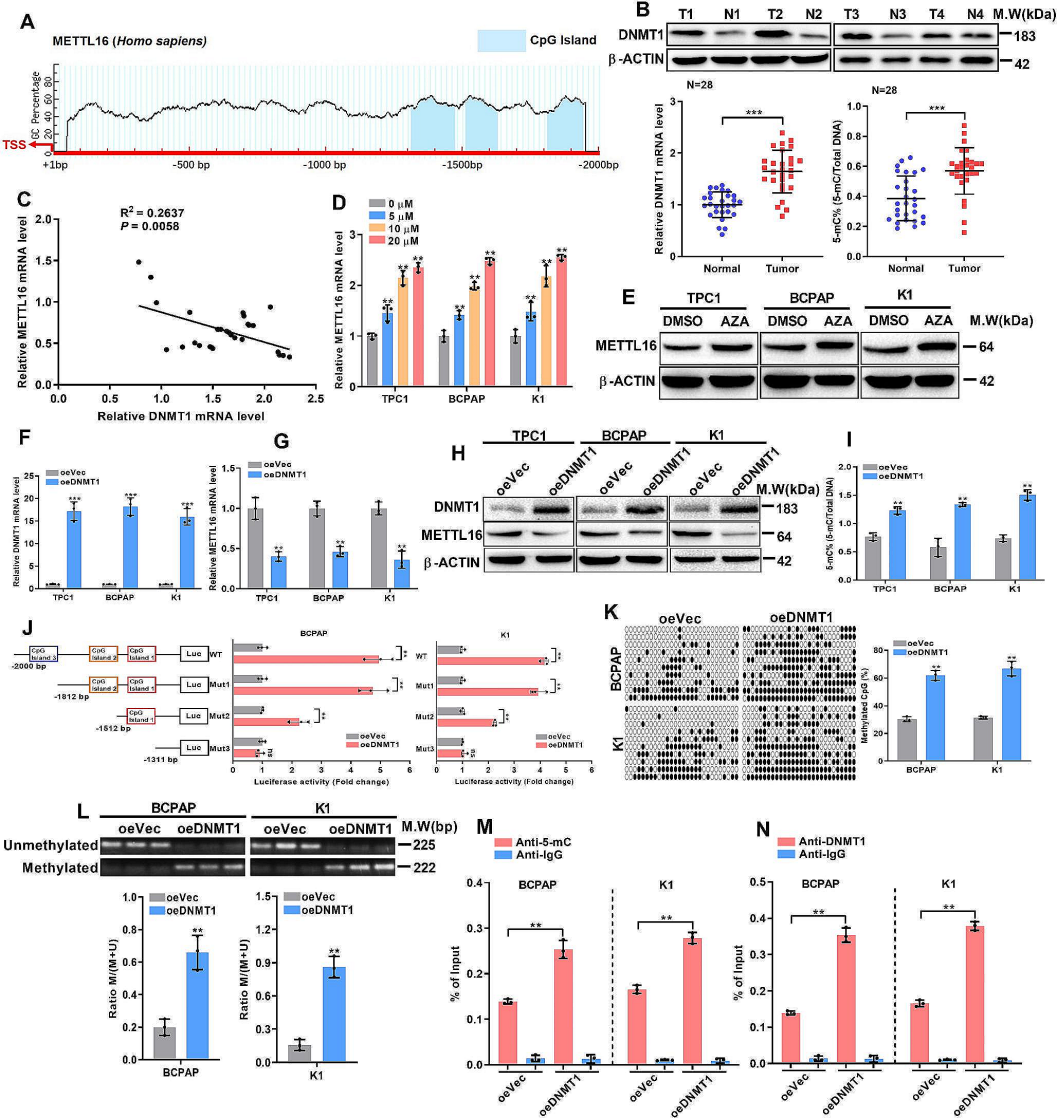
DNMT1 upregulation causes promoter hypermethylation and decreases METTL16 expression. (**A**) Methylation status analysis via the Methprimer website showed high enrichment of CpG islands in the METTL16 promoter. (**B**) mRNA levels (*n* = 28) and protein levels of DNMT1 in paired tumor tissues. The 5-mC levels were also detected in paired tumor tissues (*n* = 28). (**C**) Pearson’s correlation coefficients of METTL16 and DNMT1 in PTC tissues (*n* = 28). (D-E) METTL16 mRNA levels after treatment with different concentrations (0, 5, 10, and 20 µM) of 5-AZA for 24 h (**D**) and protein levels after treatment with 20 µM 5-AZA (**E**) in three PTC cell lines. (F–H) DNMT1 overexpression efficiency (**F**) and the effect of DNMT1 upregulation on METTL16 mRNA (**G**) and protein levels were measured (**H**) in three PTC cell lines. (**I**) 5-mC levels were detected in PTC cells with forced DNMT1 expression. (**J**) DNMT1 overexpression affected the transcription activity of METTL16 and the corresponding mutants. (**K**) Bisulfate sequencing was used to detect CpG islands in the METTL16 promoter (-1312 ~ − 1472 bp, − 1512~ − 1629 bp) in cells overexpressing DNMT1. (**L**) The methylation status of the METTL16 promoter in DNMT1-overexpressing BCPAP and K1 cells was determined using MSP. The methylation rate of the samples is presented as the ratio (M/M + U). (**M-N**) Levels of DNMT1 binding (**M**) and enrichment of 5-mC (**N**) in the METTL16 promoter in DNMT1-overexpressing BCPAP and K1 cells, as measured by ChIP‒qPCR. The data are presented as the means ± SEMs and were analyzed using Student’s t test or one-way ANOVA. *p < 0.05, **p < 0.01, ***p < 0.001. The “ns” represents non-significant

Next, in silico analysis was used to determine the mechanisms by which METTL16 was expressed at low levels in PTC tissues, and the results demonstrated a CpG island approximately − 1300 ~ − 1650 bp upstream of the METTL16 locus (Fig. 2A). DNA methylation is known to be catalyzed by DNA methyltransferase 1 (DNMT1), DNMT3A, and DNMT3B. Analysis of the mRNA levels in the collected tissue samples revealed that the DNMT1 mRNA level was significantly increased (Fig. 2B), whereas the other two genes were not significantly altered (Fig. S2A). In addition, high DNMT1 expression also induced high 5-mC levels in PTC tissues, suggesting the overexpressed DNMT1 had the catalytic activity (Fig. 2B). And qPCR analysis of PTC tissues revealed that METTL16 mRNA levels were negatively correlated with DNMT1 levels (Fig. 2C). Collectively, these data suggest that DNMT1 may regulate METTL16 through an epigenetic mechanism. To verify this hypothesis, PTC cells were treated with 5-AZA, a DNA methyltransferase inhibitor. The quantitative PCR results demonstrated an upregulation of METTL16 expression with increasing dosages (Fig. 2D), while cell viability remained unchanged (Fig. S2B). Western blot analysis also confirmed the increase in METTL16 protein levels after 5-AZA treatment (Fig. 2E). Furthermore, DNMT1 overexpression significantly decreased both METTL16 mRNA and protein levels in PTC cells (Fig. 2F-H). DNMT1 overexpression also led to an elevation in 5-mC levels, as detected by ELISA (Fig. 2I). To determine the exact mechanism by which DNMT1 regulates the 5-mC modification of METTL16, METTL16 promoter mutants were constructed, including a third CpG island (1813–1942 bp) truncation (Mut1), third and second CpG island (1513–1630 bp) truncation (Mut2), or all of the three CpG islands (the first CpG island ranged from 1313 to 1473 bp) truncation (Mut3). Data showed that DNMT1 overexpression significantly increased the luciferase activity of the WT and Mut1, and partially activated the luciferase activity of Mut2. However, Mut3 almost completely abolished this induction upon DNMT1 expression in both BCPAP and K1 cells (Fig. 2J), suggesting the first two CpG islands are essential for DNMT1-mediated METTL16 transcription. This conclusion was further verified by bisulfite sequencing PCR (BSP) and methylation-specific PCR (MSP) for CpG island methylation levels detection (Fig. 2L) in BCPAP and K1 cells. Moreover, the results of the quantitative ChIP assay revealed an increase in the binding of DNMT1 to the METTL16 promoter region (Fig. 2M) and an increase in 5-mC signals (Fig. 2N). Taken together, these results indicated that aberrant forced DNMT1 expression could induce hypermethylation of the METTL16 promoter, resulting in decreased METTL16 transcription and expression.

### METTL16 overexpression increases m^6^A levels and inhibits PTC growth

**Fig. 3:**
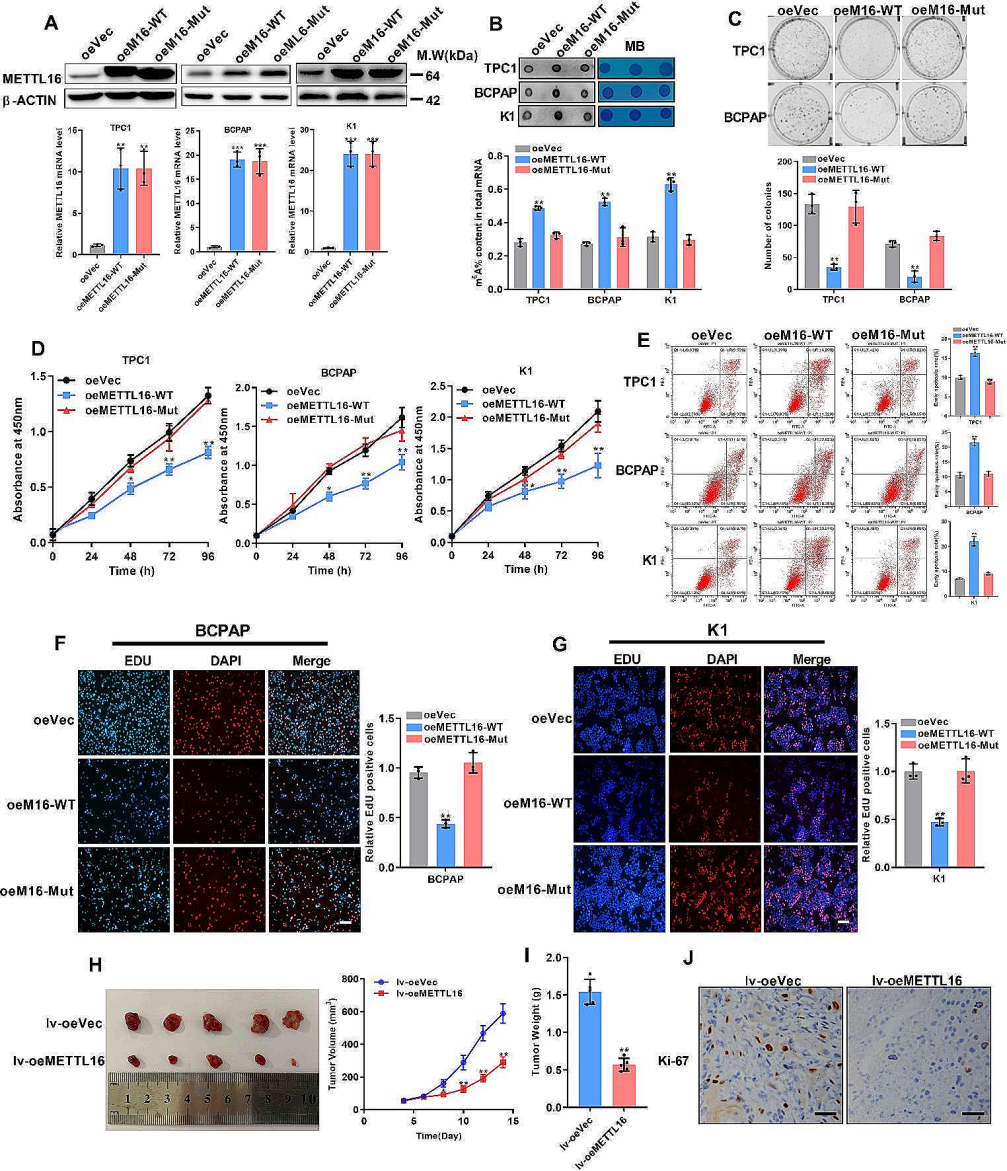
Forced METTL16 expression prohibits proliferation in PTC cells (**A**) The METTL16 overexpression efficiency was evaluated. (**B**) The m^6^A levels of total RNA isolated from wild-type or catalytic mutant METTL16-overexpressing cells were detected in PTC cells. RNAs with MB staining were used as the loading control. (C-E) Colony formation ability (**C**), cell viability (**D**), and cell apoptosis (**E**) were determined in PTC cells overexpressing wild-type or catalytically mutated METTL16. (F-G) Cell proliferation was detected by EdU staining in BCPAP cells (**F**) and K1 cells (**G**) with wild-type or catalytic mutant METTL16 overexpression; scale bars = 50 μm. (**H**) The growth of subcutaneous tumors with or without METTL16 overexpression was measured in nude mice (*n* = 5). The average tumor volume (mm^3^) of K1 cells was monitored every other day. (**I**) Tumor weights on day 15. (**J**) IHC staining of tumors for Ki-67. Scale bars = 50 μm. The data are presented as the means ± SEMs and were analyzed using Student’s t test or one-way ANOVA. *p < 0.05, **p < 0.01, ***p < 0.001

Both loss- and gain-of-function studies facilitated the evaluation of the pathological role of METTL16 in PTC. Initially, vectors for METTL16 wild-type (METTL16-WT) and METTL16 mutants (P185A/P186A) with disrupted enzymatic activity were constructed [18]. Additionally, two small interfering RNAs (siRNAs) targeting METTL16 were synthesized. The efficiency of overexpression and knockdown was confirmed in PTC cells (Fig. 3A, Fig. S3A). METTL16 overexpression resulted in increased m^6^A levels (Fig. 3B), suppressed colony formation (Fig. 3C), reduced cell viability (Fig. 3D), and promoted cell apoptosis in PTC cells (Fig. 3E). However, the overexpression of METTL16 mutants lacking m^6^A catalytic activity did not produce similar effects. In contrast, METTL16 silencing led to decreased m^6^A levels (Fig. S3B) and accelerated colony formation and cell growth but inhibited cell apoptosis in PTC cells (Fig. S3C-E). EdU assays also revealed similar effects of METTL16 on cell proliferation (Fig. 3F-G, S3F-G).

Subsequently, to understand the pathophysiological role of METTL16 in vivo, we established subcutaneous xenograft tumor models with stable METTL16 overexpression or METTL16 knockdown in BCPAP and K1 cells, where palpable tumors were formed around the 5th day. Forced METTL16 expression significantly decreased tumor growth, as indicated by decreased tumor size and weight (Fig. 3H-I), which led to a decreased level of Ki-67 (Fig. 3J). However, compared with control cells, METTL16-knockdown cells had a larger tumor size, heavier weight, and greater Ki-67 expression (Fig. S3H-J). Collectively, these results revealed that METTL16 might be a tumor suppressor in PTC through its m^6^A writer function.

### METTL16 deficiency decelerates PTC metastasis in vitro and in vivo

**Fig. 4:**
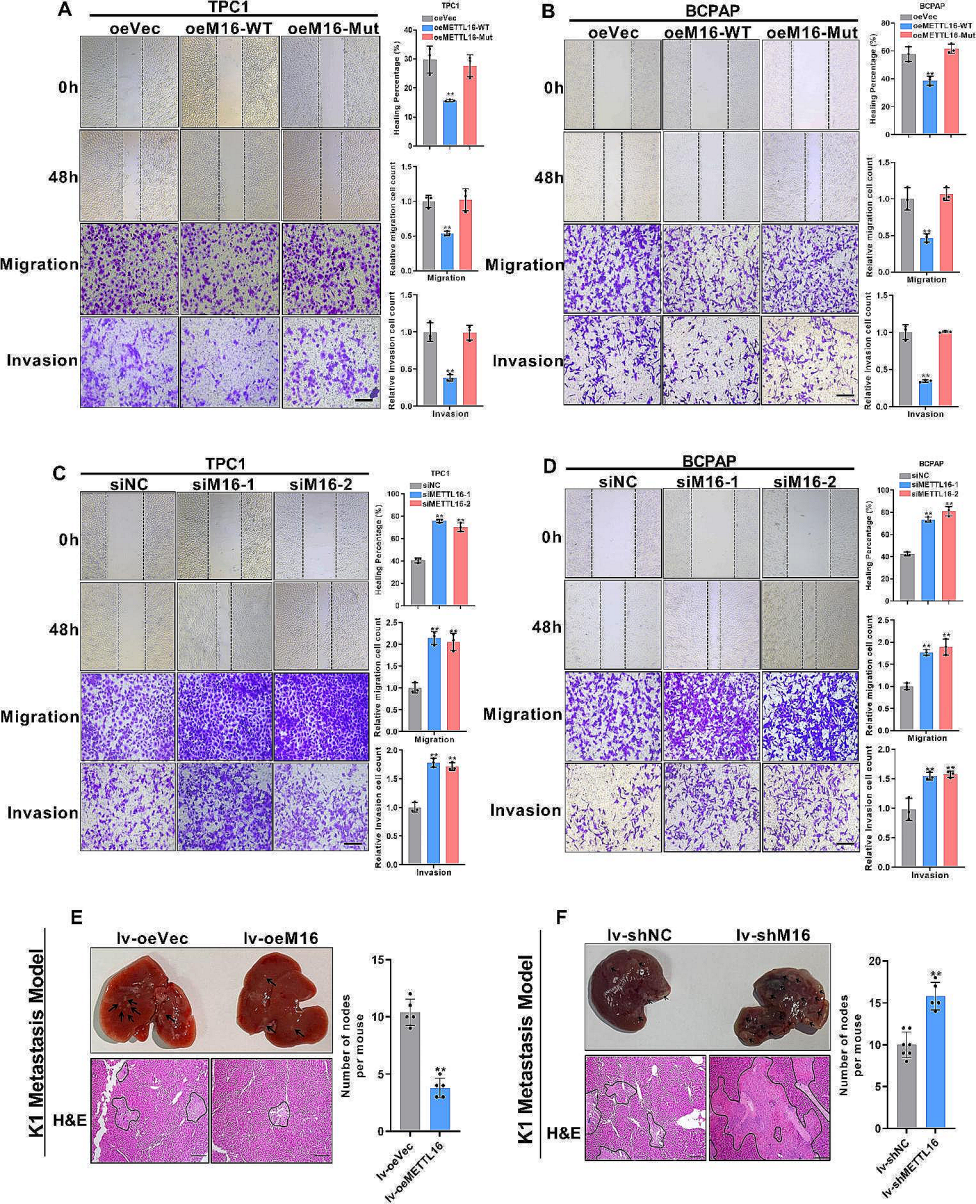
METTL16 overexpression decelerates cell metastasis. (**A-D**) Cell motility assays were performed to measure the migration and invasion of METTL16-overexpressing TPC1 cells (**A**), BCPAP cells (**B**), METTL16-knockdown TPC1 cells (**C**) and BCPAP cells (**D**). Scale bars = 50 μm. The data from the wound healing assay are shown as the quantification of the scratch width or cell number. (**E-F**) After tail vein injection of METTL16-overexpressing (**E**) or METTL16-knockdown (**F**) K1 cells, images of liver metastasis lesions were acquired, and metastatic nodules were quantified (*n* = 5). Scale bars = 50 μm. The data are presented as the means ± SEMs and were analyzed using Student’s t test or one-way ANOVA. *p < 0.05, **p < 0.01

A mobility assay was performed to assess PTC metastasis in cells with METTL16 overexpression or knockdown. The results revealed that METTL16-WT overexpression markedly increased the migration distance of TPC-1 and BCPAP cells; however, overexpression of the catalytic mutant METTL16 did not increase the migration distance (Fig. 4A-B). In the Transwell assay, METTL16-WT, but not METTL16-Mut, obviously reduced TPC-1 and BCPAP cell migration and invasion (Fig. 4A-B). In contrast, METTL16 knockdown had the opposite effect on TPC-1 and BCPAP cells (Fig. 4C-D). Next, an in vivo liver metastasis model was established by injecting K1 cells overexpressing METTL16 and the corresponding control cells via the tail vein. METTL16 overexpression markedly inhibited tumor liver metastasis, as measured by the size and number of nodules in the liver (Fig. 4E). Conversely, METTL16 knockdown in K1 cells promoted PTC liver metastasis (Fig. 4F). In conclusion, these findings reveal that METTL16 could inhibit PTC metastasis.

### METTL16 attenuates lipid metabolism via m^6^A-mediated stabilization of SCD1 mRNA

**Fig. 5:**
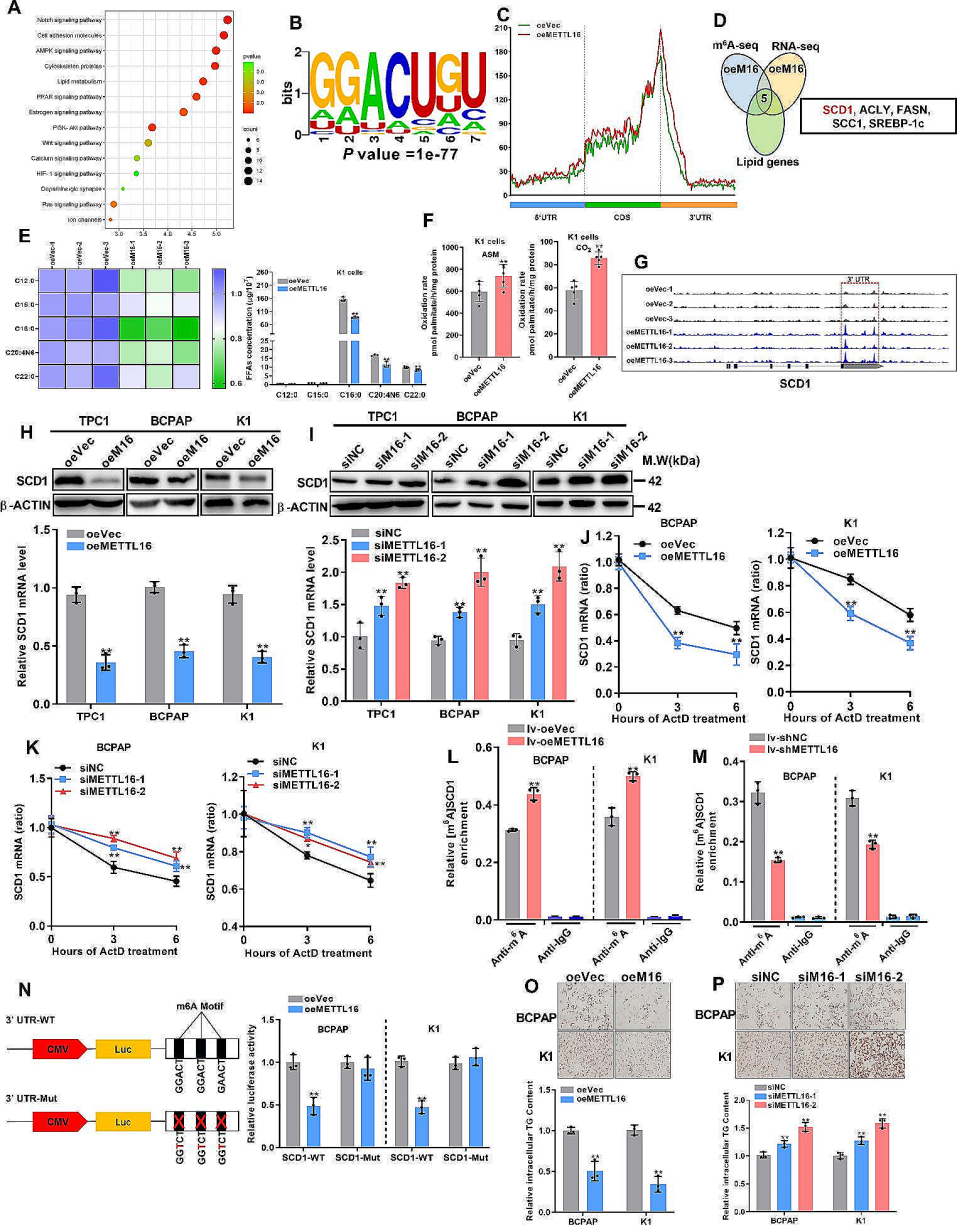
METTL16 attenuates lipid metabolism via m^6^ A-mediated stabilization of SCD1 mRNA. (**A**) RNA-seq analysis of METTL16-overexpressing K1 cells. (**B**) The m^6^A-seq data revealed that GGACU was the predominant motif in METTL16-overexpressing K1 cells. (**C**) The m^6^A peak distribution in the mRNA transcripts of K1 cells with upregulated METTL16 expression. (**D**) RNA-seq and m^6^A-seq were used to filter five lipid metabolism-related genes in K1 cells overexpressing METTL16. (**E**) FFA production in K1 cells with upregulated METTL16 expression was measured using GC‒MS. (**F**) Palmitate oxidation rate (partial and complete oxidation) in K1 cells with METTL16 overexpression. (**G**) m^6^A sites in SCD1 mRNA were identified through m^6^A-seq. (**H-I**) The expression levels of SCD1 were determined in K1 cells with METTL16 overexpression (**H**) or METTL16 restraint (**I**). (**J-K**) Stability of SCD1 mRNA in cells with forced METTL16 expression (**J**) or METTL16 restraint expression (**K**) BCPAP and K1 cells treated with 5 µg/mL actinomycin D. (**L-M**) RIP-qPCR analysis of SCD1 m^6^A levels in METTL16 overexpression (**L**) or METTL16 knockdown (**M**) BCPAP and K1 cells. (**N**) Luciferase activity in METTL16-overexpressing cells co-transfected with the SCD1 3’UTR with or without the A-to-T mutation. (**O-P**) Cellular neutral lipids were stained with Oil Red O in BCPAP and K1 cells with METTL16 overexpression (**O**) or knockdown (**P**). Scale bars = 50 μm. The data are presented as the means ± SEMs and were analyzed using Student’s t test or one-way ANOVA followed by the Newman‒Keuls multiple comparison test. *p < 0.05, **p < 0.01

RNA-seq data indicated that METTL16 overexpression affects lipid metabolism, suggesting that METTL16 might be involved in PTC progression via its m^6^A writer function (Fig. 5A). To verify this assumption, m^6^A-seq was first performed in METTL16-overexpressing K1 cells. The consensus motif GGACU was highly enriched (Fig. 5B), and the m^6^A peak located around the stop codon was elevated in response to METTL16 overexpression (Fig. 5C). By combining the hypermethylated m^6^A peaks with the differentially expressed lipid genes, five genes were ultimately screened out (Fig. 5D), and SCD1 was the most significantly downregulated gene (fold change = 0.36, *P* = 1.58 × 10^–4^) (Fig. S4A-B). In addition, the Gas Chromatography-Mass Spectrometer (GC‒MS) assays showed that METTL16 overexpression markedly reduced the abundance of a series of free fatty acids (FFAs), including palmitate and oleic acid (Fig. 5E), and increased fatty acid b-oxidation (Fig. 5F) in K1 cells. In contrast, METTL16 knockdown in K1 cells significantly increased FFA abundance (Fig. S4C) and reduced fatty acid b-oxidation (Fig. S4D). Furthermore, METTL16 overexpression increased the m^6^A peak density in the 3’UTR of SCD1 mRNA (Fig. 5G). These findings indicate that METTL16 may be involved in PTC progression by modulating SCD1 expression via m^6^A modification and subsequently regulating lipid metabolism and PTC cell proliferation and metastasis. To test this conclusion further, the effects of METTL16 on SCD1 expression were evaluated. The data showed that forcing METTL16 expression decreased SCD1 expression (Fig. 5H), but suppressing METTL16 had the opposite effect (Fig. 5I). Considering that m^6^A modification is correlated with the stability of mRNAs, an RNA stability assay was performed. qRT‒PCR revealed that METTL16 overexpression decreased SCD1 mRNA stability (Fig. 5J), while the opposite effect (Fig. 5K) was observed in METTL16-silenced BCPAP and K1 cells. Similar results were obtained for the TPC1 cells (Fig. S4E). Furthermore, a methylated RNA immunoprecipitation (MeRIP-qPCR) assay demonstrated that METTL16 overexpression led to an increase in the m^6^A abundance of SCD1 (Fig. 5L), whereas METTL16 silencing resulted in a decrease in its m^6^A abundance (Fig. 5M) in BCPAP and K1 cells. Similar results were also observed in TPC1 cells (Fig. S4F-G).

Moreover, the website tool SRAMP (http://www.cuilab.cn/sramp) revealed three putative m^6^A modification sites in the SCD1 3’UTR (Fig. S4H). To confirm that METTL16 regulates SCD1 expression via m^6^A modification, an SCD1 3’ UTR containing an A-to-T point mutation was constructed for use as a dual-luciferase reporter (pmirGlo). METTL16 overexpression reduced the reporter activity of only the wild-type, not the mutant, type, indicating the crucial role of METTL16-regulated m^6^A methylation in regulating SCD1 expression (Fig. 5N). Palmitic acids can be transformed into oleic and palmitoleic acids mainly by SCD1, which promotes intracellular triglyceride (TG) synthesis. Hence, the TG content was ultimately measured. The TG content assay showed that METTL16 overexpression decreased cellular TG accumulation in PTC cells (Fig. 5O), while METTL16 knockdown had the opposite effect (Fig. 5P). In conclusion, these results indicate that the METTL16/m^6^A/SCD1 axis plays a role in regulating PTC progression through the modulation of lipid metabolism.

### METTL16 regulates the mRNA stability of SCD1 via YTHDC2

**Fig. 6:**
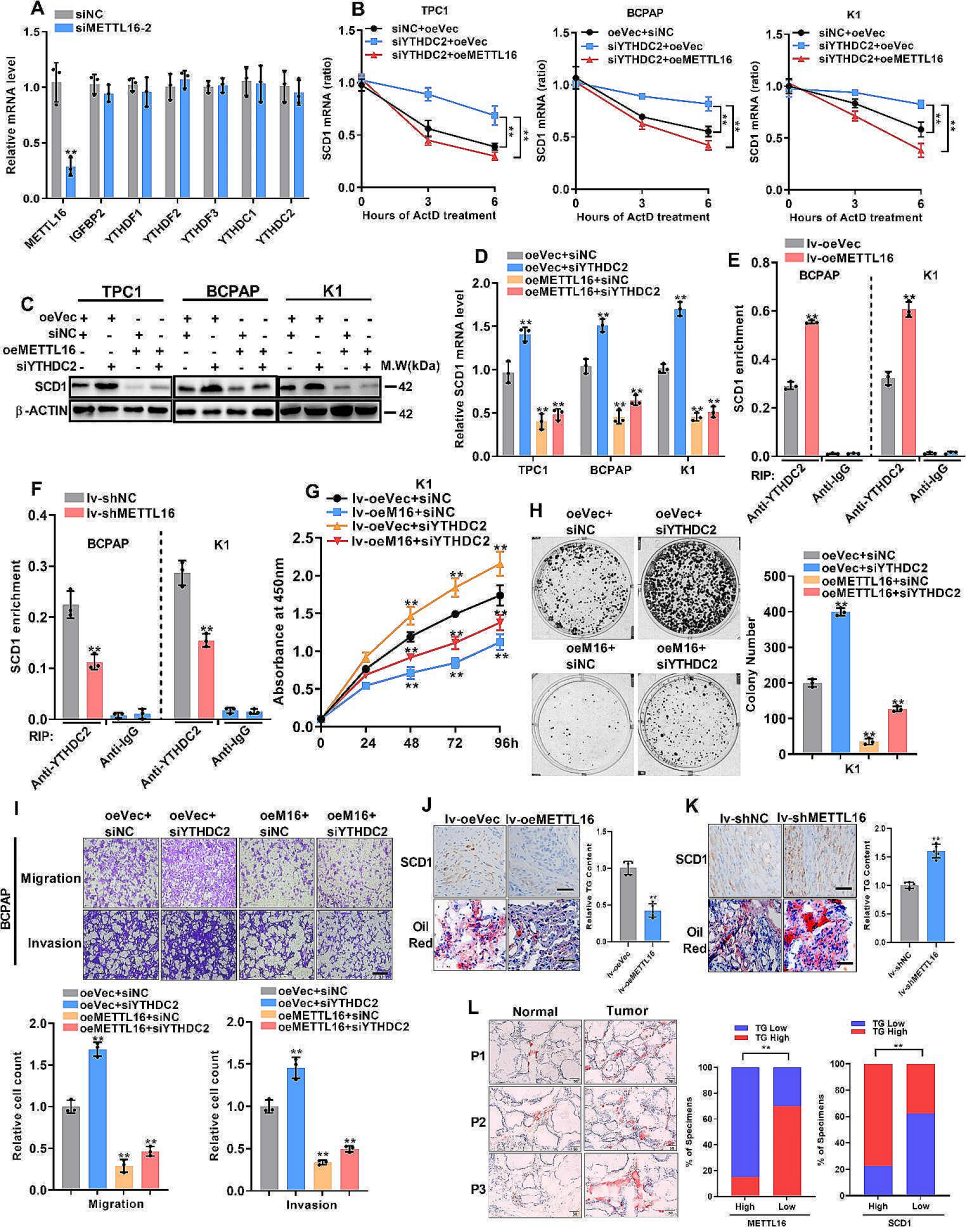
METTL16 regulates the mRNA stability of SCD1 via YTHDC2. (**A**) The mRNA levels of m^6^A readers after METTL16 knockdown. (**B**) Decay in SCD1 mRNA expression in three PTC cell lines overexpressing METTL16 in combination with YTHDC2 knockdown by siRNA. (**C-D**) Expression levels of SCD1 in PTC cells overexpressing METTL16 and with YTHDC2 knockdown. (**E-F**) The RIP-qPCR assay with the YTHDC2 antibody revealed that the binding of YTHDC2 to SCD1 mRNA could be affected by METTL16 expression (**E**) or METTL16 deletion (**F**) in BCPAP and K1 cells. IgG was used as the negative control. (**G-I**) Cell proliferation and motility analysis of PTC cells transfected with the indicated vector or siRNAs. (**J-K**) Sections of METTL16-overexpressing (**J**) or METTL16-knockdown (**K**) tumors were stained with anti-SCD1 antibodies and Oil Red O; scale bars = 50 μm. The TG content was also measured. (**L**) Correlations of METTL16 and SCD1 expression with TG content in human PTC specimens (*n* = 58). The data are presented as the means ± SEMs and were analyzed using Student’s t test or one-way ANOVA. *p < 0.05, **p < 0.01

m^6^A functions in gene expression primarily through different m^6^A readers. Moreover, SCD1 was negatively regulated by m^6^A methylation. Therefore, the m^6^A reader protein that promotes mRNA decay via m^6^A modification should be involved in METTL16-mediated SCD1 expression. YTHDC2 might be a candidate because, first, METTL16 inhibition had no effect on different m^6^A regulators (Fig. 6A). Furthermore, a prior study demonstrated that YTHDC2 could reduce the stability of SCD1 mRNA in the liver [19], and YTHDC2 exhibited lower expression levels in PTC tissues (Fig. S5A-B). Addition [20]ally, the expression pattern of YTHDC2 showed a positive correlation with METTL16 expression (Fig. S5C-D). Consequently, the influence of YTHDC2 on METTL16-regulated SCD1 mRNA stabilization was investigated. Deletion of YTHDC2 extended or partially extended the lifespan of PTC cells or PTC cells with METTL16 overexpression, stratified by SCD1 mRNA expression (Fig. 6B). Furthermore, YTHDC2 deletion enhanced SCD1 expression, but this effect was nullified in METTL16-overexpressing PTC cells (Fig. 6C-D). Using a YTHDC2 antibody, a methylated RNA immunoprecipitation assay indicated that YTHDC2 markedly enriched SCD1 mRNA, and METTL16 overexpression enhanced the interaction between YTHDC2 and SCD1 mRNA in BCPAP and K1 cells (Fig. 6E). Nevertheless, METTL16 knockdown diminished this interaction (Fig. 6F). To further confirm that the tumor inhibitory effects of METTL16 were associated with YTHDC2, cell proliferation and metastasis were assessed. The data indicated that the carcinogenesis of PTC cells inhibited by YTHDC2 was restored by METTL16 overexpression (Fig. 6G-I). Moreover, SCD1 expression and TG accumulation were decreased in the K1 tumor xenograft model overexpressing METTL16 (Fig. 6J) but increased with METTL16 knockdown (Fig. 6K). Consistent with these findings, after we divided the tissues into METTL16- or SCD1-high or -low groups, METTL16 expression was negatively correlated with TG content in PTC tissues, while SCD1 expression was positively correlated with TG content in those tissues (Fig. 6L). These findings indicate that YTHDC2 functions as an m^6^A reader in METTL16-mediated SCD1 expression and aids METTL16 in the regulation of lipid metabolism and PTC progression.

### The SCD1 inhibitor A939572 is a potential therapeutic agent for METTL16-mediated PTC progression

**Fig. 7:**
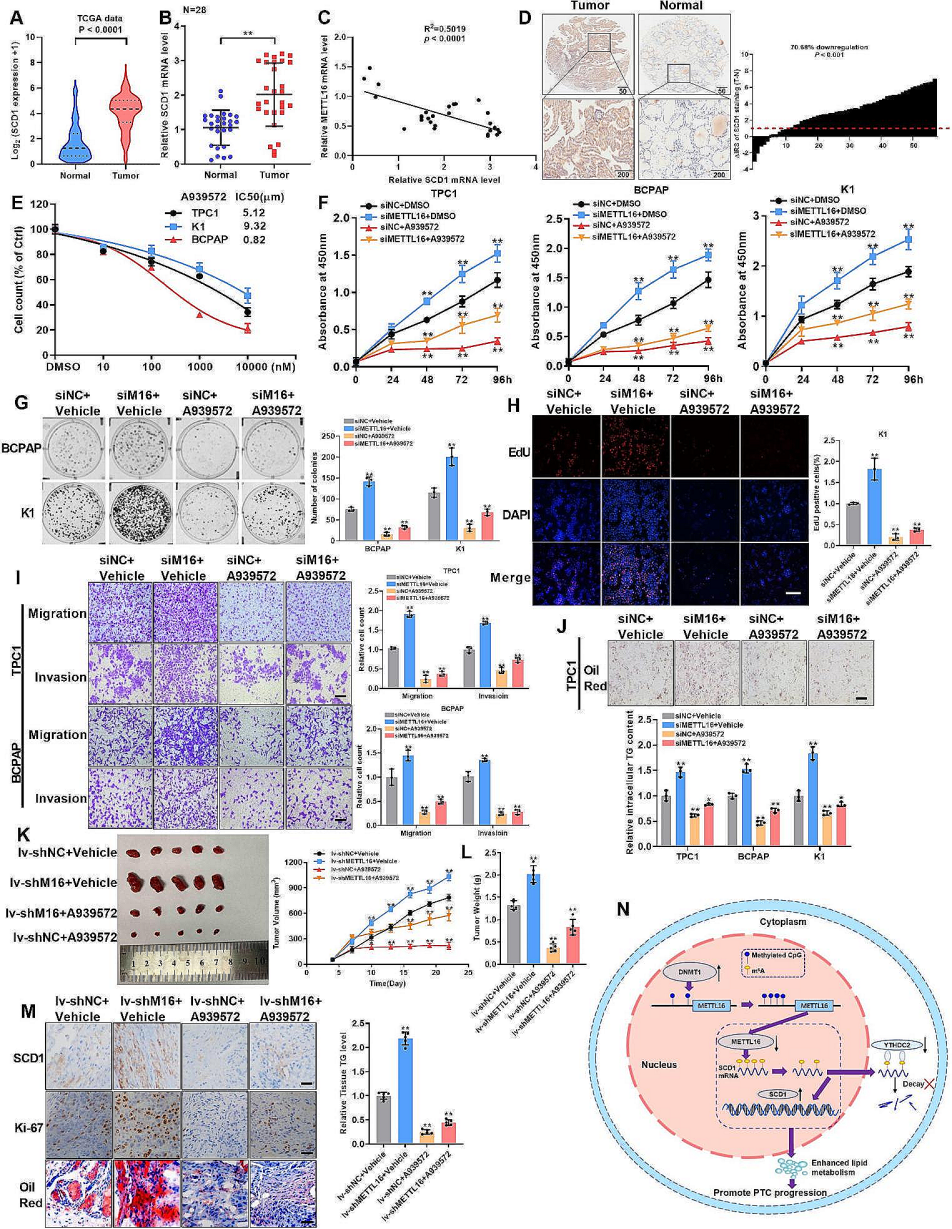
The SCD1 inhibitor A939572 is a potential therapeutic agent for METTL16-mediated PTC progression. (**A**) The TCGA database showed differential SCD1 expression in normal (*n* = 178) and tumor tissues (*n* = 493). (**B**) The mRNA levels of METTL16 in 28 paired PTC tissues. (**C**) Pearson’s Pearson correlation between SCD1 and METTL16 in PTC tissues (*n* = 28). (**D**) Representative IHC images of the tissue microarray incubated with an SCD1 antibody. Scale bars = 50 or 200 μm. (**E**) Proliferative dose response of PTC cells to the SCD1 inhibitor A939572. (**F-G**) Effects of A939572 on (**F**) cell viability and (**G**) colony formation in PTC cells with METTL16 knockdown. (**H**) The results of the EdU incorporation assay indicating cell proliferation were similar to those of A939572 in K1 cells with METTL16 knockdown. (**I**) Effect of A939572 on cell motility. (**J**) Measurement of TG content in PTC cells with METTL16 deletion and after A939572 treatment. (**K-L**) Tumor growth rate (**K**) and tumor weight (**L**) in mice treated with METTL16 inhibition and A939572 (*n* = 5). (**M**) Sections of tumor xenografts from mice with METTL16 knockdown or METTL16 knockdown with A939572 were stained with antibodies against SCD1 and ki-67 for IHC. Scale bars = 50 μm. The data are presented as the means ± SEMs and were analyzed using Student’s t test or one-way ANOVA. *p < 0.05, **p < 0.01

To assess the impact of pharmacological inhibition of SCD1 on METTL16-mediated PTC progression, the clinical relevance of SCD1 in PTC tissue samples was examined. Data showed that PTC patients exhibiting high SCD1 expression had lower survival rates (Fig. S5E). And both TCGA and RT‒qPCR data revealed increased expression of SCD1 in PTC tissues (Fig. 7A-B) and a negative correlation with METTL16 (Fig. 7C). In addition, the IHC staining of the tissue microarray yielded similar results (Fig. 7D). Next, the SCD1 inhibitor A939572, known for its documented ability to inhibit the growth and metastasis of various tumor cells, was selected to evaluate its therapeutic impact on PTC cells. The proliferation of all three PTC cell lines decreased with increasing dosage (Fig. 7E), especially at the 50% inhibitory concentration (IC_50_) of BCPAP in the low nanomolar dose range (820 nM). After PTC cells were treated with an IC_50_ of A939572, proliferation and colony formation were significantly inhibited, and the EdU intensity was reduced by METTL16 knockdown (Fig. 7F-H). In addition, A939572 reversed the effects of METTL16 downregulation on the motility of both TPC1 and BCPAP cells (Fig. 7I). TG accumulation was also reversed by A939572 (Fig. 7J). Subsequent xenograft tumorigenesis revealed that, in both groups treated with A939572, the tumors grew less rapidly than those in the vehicle alone group (Fig. 7K-L). IHC and Oil Red O staining demonstrated that METTL16 knockdown increased SCD1, Ki-67, and TG staining in K1 tumor xenografts but decreased this parameter in cells treated with A939572 (Fig. 7M). Collectively, these findings substantiate the therapeutic potential of A939572 in addressing PTC progression mediated by the METTL16/SCD1 axis.

## Discussion

The present study examined a series of m^6^A modulators and revealed that the m^6^A writer METTL16, which is involved in decreased m^6^A modification in PTCs, was expressed at lower levels in PTC tissues than in normal tissues. Low METTL16 expression was associated with poor prognosis in PTC patients. However, the introduction of forced METTL16 expression could alleviate these malignant symptoms, indicating its role as an anti-tumorigenic factor in PTC. DNMT1-mediated DNA hypermethylation within the METTL16 promoter region was responsible for the reduction in METTL16 transcription and expression levels. Decreased METTL16 expression also led to a diminished m^6^A modification of SCD1 mRNA. Consequently, the inability of YTHDC2, an m^6^A reader, to bind to the m^6^A site on SCD1 mRNA disrupted its degradation process, resulting in elevated SCD1 expression. This, in turn, enhanced lipid metabolism in PTC cells, thereby facilitating PTC progression and leading to a poorer clinical prognosis (Fig. 7N). In summary, METTL16 may serve as a potential predictive molecular marker for both PTC diagnosis and prognosis evaluation.

METTL16 has been extensively investigated [21], with numerous studies documenting its involvement in cell proliferation, metastasis [22], and chemotherapy [23] in various cancer types. Nonetheless, the underlying cause of aberrant METTL16 expression remains enigmatic. In this study, we initially identified that DNMT1 overexpression-induced hypermethylation inhibits METTL16 transcription, resulting in decreased METTL16 expression in PTC. Furthermore, a prior study reported that DNMT1 overexpression can promote PTC progression through CpG methylation [24] and DNMT1 could response to oncogenic signals that are mediated by the Ras-c-Jun oncogenic signaling pathway [25] and both Ras and c-Jun were activated in PTC [26, 27]. This is why DNMT1 is transcriptionally upregulated in PTC. These findings robustly support the significant epigenetic control of METTL16 by DNMT1 in PTC. However, whether DNMT1-mediated METTL16 expression is also observed in other cancer types warrants further investigation.

By analyzing m^6^A-seq data, we found that SCD1 was the target of METTL16. SCD1 has been reported to be involved in many cancers [28], including lung cancer [20], gastric cancer [29], and PTC [30]. Our study shows that METTL16’s antitumor function is linked to elevated m^6^A levels, leading to the direct destabilization of SCD1 mRNA. This observation suggests the potential involvement of an mRNA degradation regulator in METTL16-mediated SCD1 expression. RNA stability is primarily governed by m^6^A reader proteins [31], and among these proteins, YTHDC2 emerges as a promising candidate [32]. The reasons are as follows. First, YTHDC2 has been reported to recognize m^6^A methylation sites on lipogenic genes and bind to mRNAs, including SCD1 mRNA, to reduce their stability [19]. In addition, YTHDC2 is expressed at lower levels in PTC tissues than in normal tissues [12], and forced YTHDC2 expression prevents PTC progression [33]. The current investigation has further unveiled that YTHDC2 serves as a tumor suppressor in PTC. In summary, our findings establish that METTL16 overexpression can induce the degradation of SCD1 mRNA through the involvement of YTHDC2 in PTC. However, whether METTL16 demethylation induces additional effects on PTC progression is largely elusive.

High lipogenicity is a well-known characteristic phenotype of tumor cells because of the aberrantly high demand for nutrients [14, 34]. Studies have unveiled that m^6^A-dependent RNA methylation intricately intersects with lipid metabolism in diverse cancer cell types [35]. Furthermore, SCD1 is a target of METTL16 and has the capability to convert palmitic acid into monounsaturated fatty acids, which are preferentially transformed into TG [36]. These pieces of evidence strongly indicate a potential link between the RNA methyltransferase METTL16 and lipid metabolism in PTC. Our study demonstrates that METTL16 restrains the production of palmitic acids and oleic acids in PTC cells. Additionally, it was observed that METTL16 inhibits TG synthesis, implying that the disrupted lipid metabolism in PTC may be triggered by METTL16 downregulation-induced SCD1 overexpression. Moreover, individuals with a higher body mass index (BMI) and obesity exhibit an elevated incidence of thyroid cancer [15], hinting at the possibility that increased FFA synthesis may be utilized by normal thyroid cells with low METTL16 expression, ultimately contributing to PTC development.

The SCD1 inhibitor A939572, a potential antitumor molecular drug [37], restored lipogenic activity and significantly decreased proliferation, migration, invasion and lipid metabolism in forced METTL16-expressing PTC cells. Therefore, inhibiting METTL16 expression and increasing SCD1 expression in PTC might constitute a new therapeutic strategy for the treatment of PTC. Notably, the IC_50_ of A939572 in TPC1 and K1 cells was high (4–8 µM). For SCD1 inhibitors to be effective, the combination of A939572 with chemical drugs could be effective. In addition, developing new drugs targeting METTL16-mediated SCD1 upregulation might considerably benefit PTC patients.

In summary, we have elucidated the oncogenic role of METTL16 in the progression of PTC. Mechanistically, the METTL16/m^6^A-YTHDC2/SCD1 axis appears to be implicated in the tumorigenesis and metastasis of PTC through the regulation of lipid metabolism. Our findings could lead to the development of new PTC therapeutic options by inhibiting the METTL16 pathway in combination with SCD1 inhibition.

### Electronic supplementary material

Below is the link to the electronic supplementary material.

Supplementary Material 1

## Data Availability

All data generated or analyzed during this study are available from the corresponding author on reasonable request.

## Abbreviations

PTC: Papillary thyroid cancer
TCGA: The Cancer Genome Atlas
OS: Overall survival
IHC: Immunohistochemistry
TNM: Tumor node metastasis
AZA: 5-Aza-2’-deoxycytidine
5-mC: 5-methylcytosine
BSP: Bisulfate sequencing
MSP: Methylation-specific PCR
ChIP: Chromatin immunoprecipitation
EdU: 5-ethynyl-2’–deoxyuridine
GC-MS: Gas chromatography-mass spectrometry
RIP: RNA immunoprecipitation
TG: Triglyceride
siRNA: Small interfering RNA
ActD: Actinomycin D
IHC: Immunohistochemistry
lv-shM16: Lentivirus – short hairpin RNA against METTL16
lv-oeM16: Lentivirus – METTL16 overexpression vector. lv-shNC,lentivirus – short hairpin RNA against negative control
lv-oeVec: Lentivirus – empty vector
MB: Methylene Blue
DAPI: 4′,6-diamidino-2-phenylindole
ASM: Acid-soluble metabolites
