# Supplementary material for: METTL16 inhibits papillary thyroid cancer tumorigenicity through m6A/YTHDC2/SCD1-regulated lipid metabolism
Source: Cell Mol Life Sci. 2024 Feb 9;81(1):81. doi: 10.1007/s00018-024-05146-x (PMC10857971; doi:10.1007/s00018-024-05146-x)
Supplement: Supplementary file 1 — Supplementary Material 1 [file 18_2024_5146_MOESM1_ESM.docx]

**Supplementary figure legends**


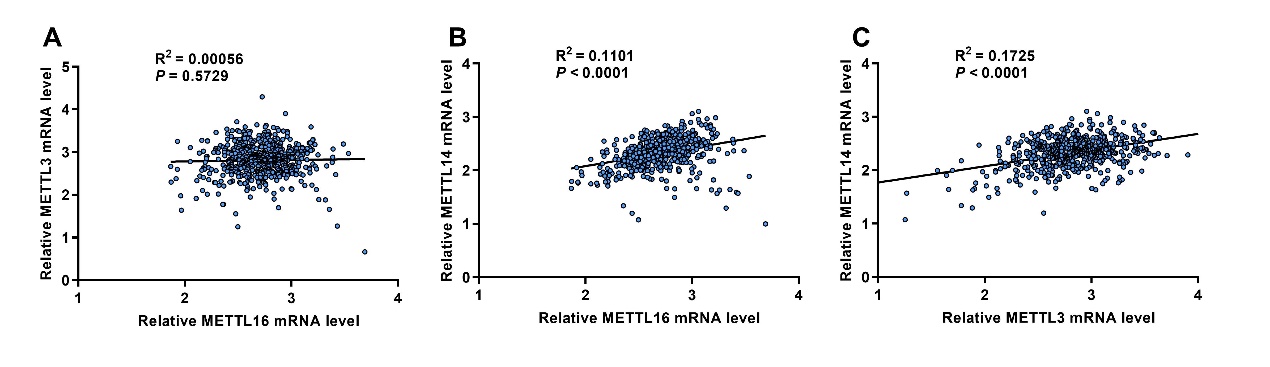


Fig. S1. METTL14 positively correlated with METTL16 in PTC tissues. (A-C) The correlation between (A) METTL3 and METTL16, (B) METTL14 and METTL16, (C) METTL3 and METTL14 in TCGA database.


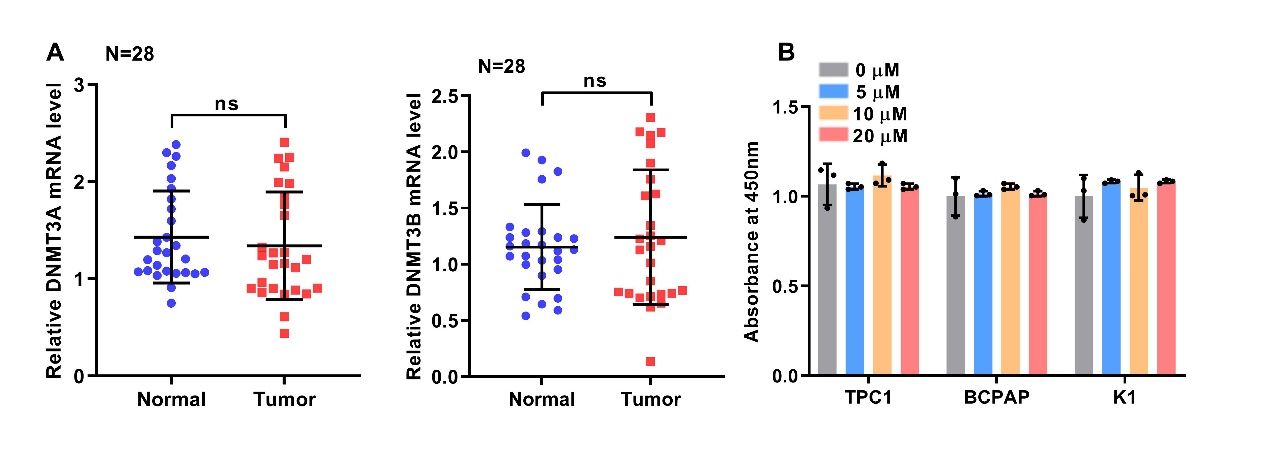


Fig. S2. Effect of AZA on cell viability. (A) The expression discrepancy of DNMT3A and DNMT3B in paired tissues (n=28). (B) Cell viability after different concentration of AZA treatment (0, 5, 10 20 μM). Data are represented as the mean ± SEM. The “ns” represents non-significant.


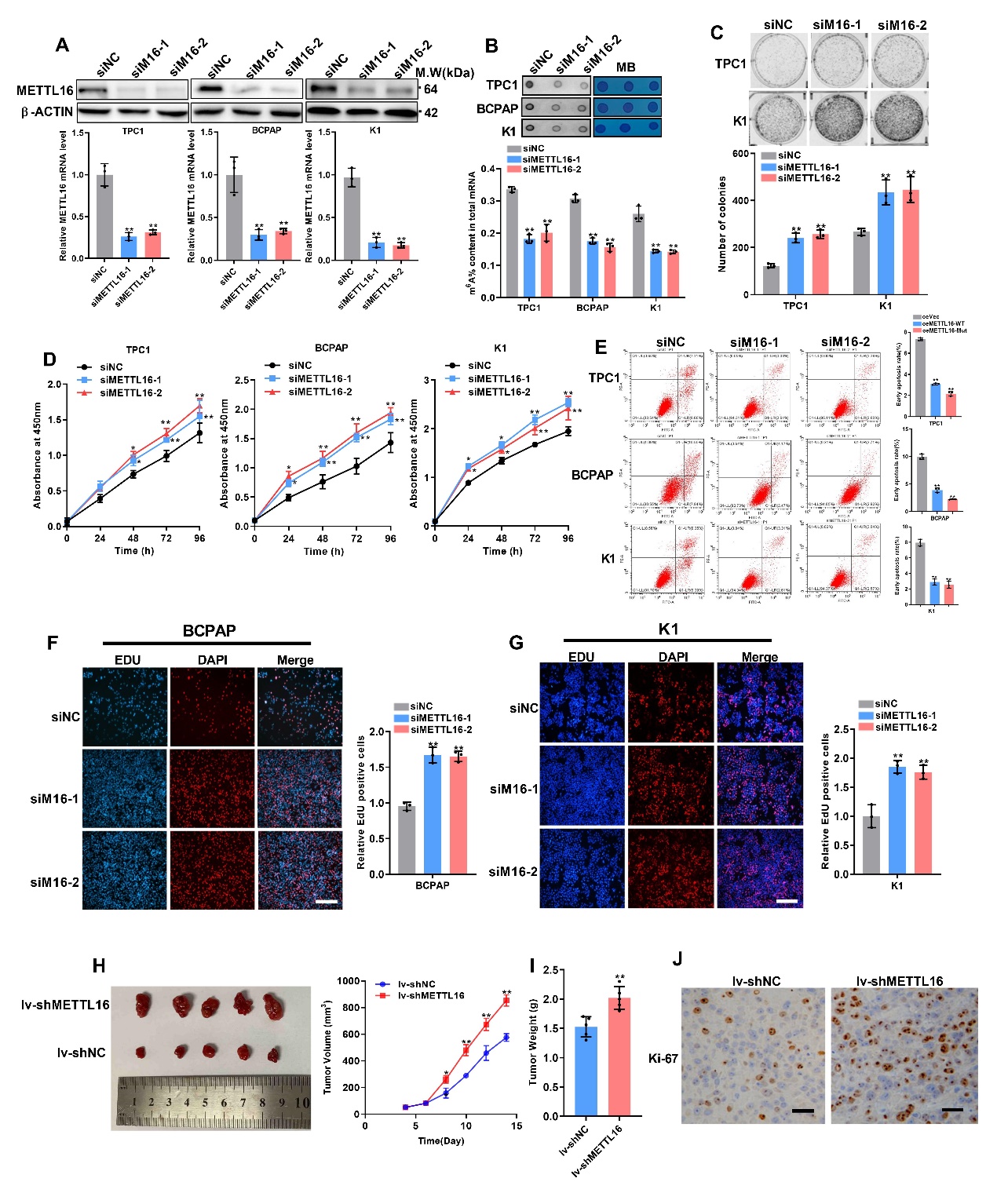


Fig. S3. METTL16 knockdown promotes proliferation in PTC cells. (A) The METTL16 knockdown efficiency was measured. (B) The m^6^A levels of total RNA isolated from METTL16-knockdown were detected in PTC cells. RNAs with MB staining were the loading control. (C-E) The colony formation (C), cell viability (D), and cell apoptosis (E) were determined in PTC cells with METTL16 knockdown. (F-G) The cell proliferation was detected by EdU in BCPAP cells (F) or K1 cells (G) with METTL16 deletion, Scale bars = 50 µm. (H) The growth of subcutaneous tumors with METTL16 knockdown was measured in nude mice (n = 5). Average tumor volume (mm^3^) of K1 cells was monitored every other day. (I) Tumors weight at day 15. (J) IHC stains of tumors with Ki-67. Scale bars = 50 µm. Data are represented as mean ± SEM. **P* < 0.05, ** *P* < 0.01 *vs* control group.


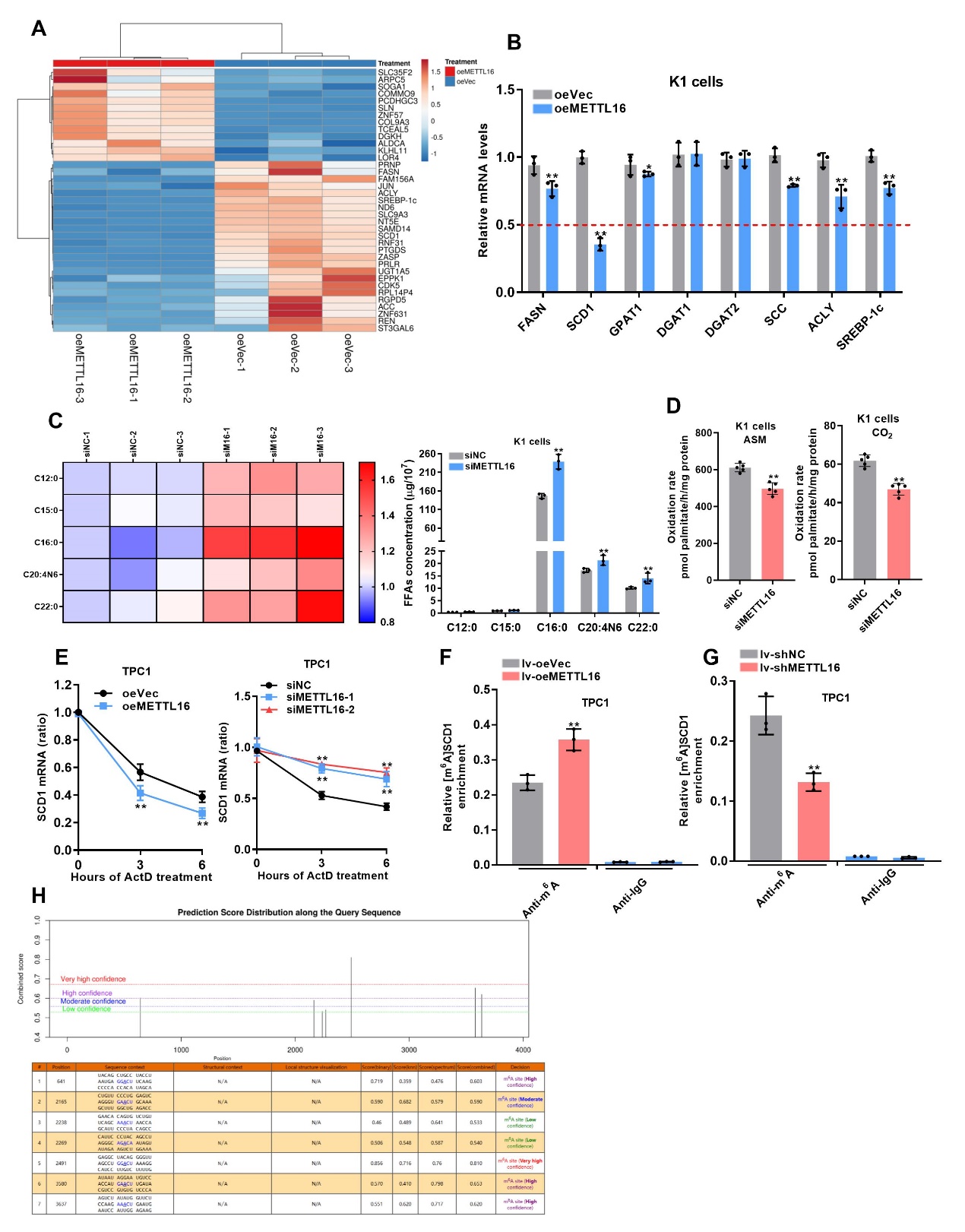


Fig. S4. METTL16 attenuates lipid metabolism via m^6^A-mediated stability of SCD1 mRNA. (A) RNA-seq data of lipid genes change after METTL16 overexpression. (B) qPCR determination of lipid genes in K1 cells with METTL16 overexpression. (C) The FFAs production in K1 cells with METTL16 knockdown was measured using GC-MS. (D) Palmitate oxidation rate (partial and complete oxidation) in K1 cells with METTL16 knockdown. (E) Stability of SCD1 mRNA in forced METTL16 expression or METTL16 restraint expression TPC1 cells. (F-G) RIP-qPCR analysis of SCD1 m^6^A levels in METTL16 overexpression (F) or METTL16 knockdown (G) TPC1 cells. (H) The putative m^6^A sites in SCD 3’UTR predicted by SRAMP. The “Decision” with high or very high confidence would be taken into consideration (Position 641, 2491 and 3580). Data are represented as mean ± SEM. **P* < 0.05, ** *P* < 0.01 *vs* control group.


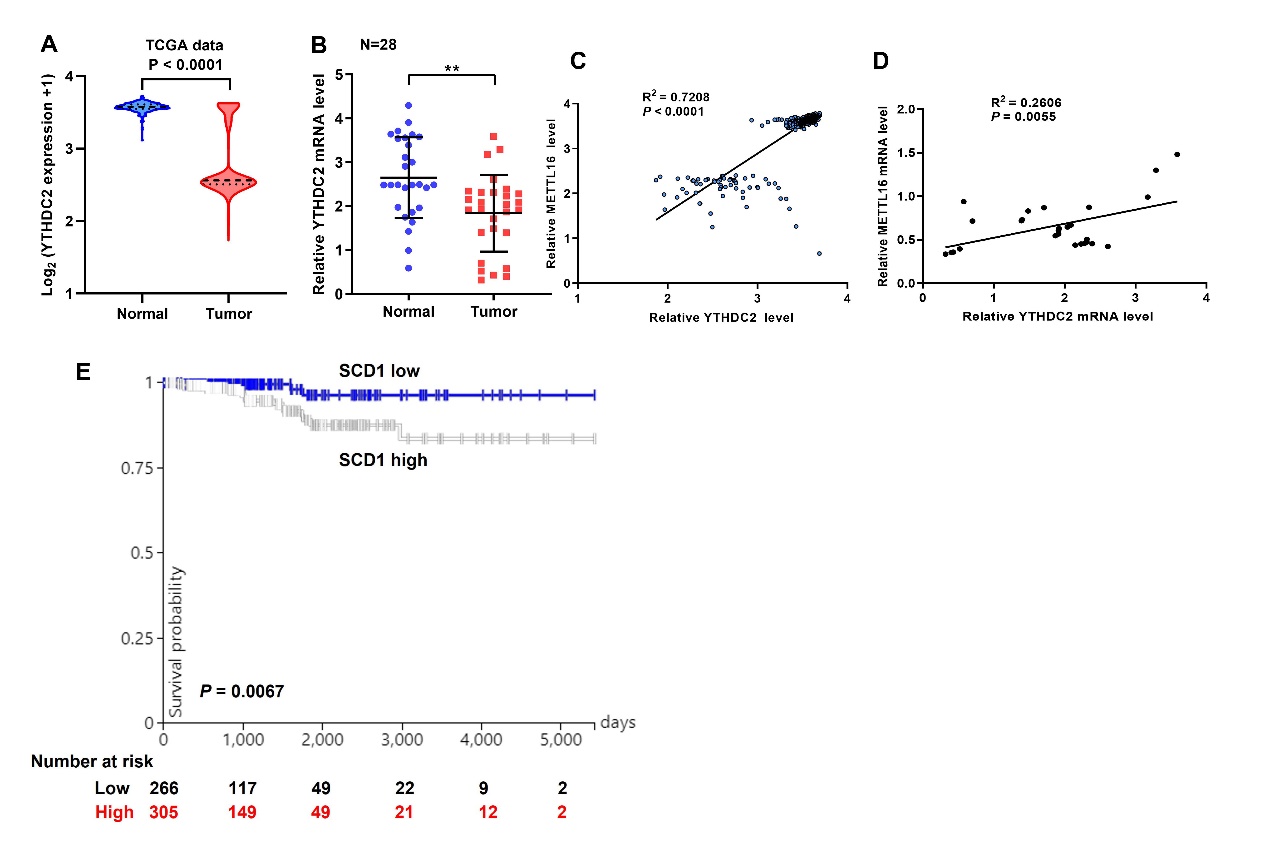


Fig. S5. The expression pattern of YTHDC2 in PTC tissues. (A) Expression of YTHDC2 in normal thyroid tissues (n = 178) and PTC tissues (n = 493) in TCGA. (B) Levels of YTHDC2 mRNA in paired PTC tissues (n = 28). (C-D) Correlation of YTHDC2 and METTL16 in TCGA and collected paired PTC tissues. (E) Kaplan‒Meier survival curves of PTC patients in the TCGA cohort. Data are represented as mean ± SEM. **P* < 0.05, ** *P* < 0.01 *vs* control group.
